# Estimation of Overspread Underwater Acoustic Channel Based on Low-Rank Matrix Recovery

**DOI:** 10.3390/s19224976

**Published:** 2019-11-15

**Authors:** Jie Li, Fangjiong Chen, Songzuo Liu, Hua Yu, Fei Ji

**Affiliations:** 1 School of Electronic and Information Engineering, South China University of Technology, Guangzhou 510640, Chinaeefjchen@scut.edu.cn (F.C.);; 2 Acoustic Science and Technology Laboratory, Harbin Engineering University, Harbin 150001, China; 3 Key Laboratory of Marine Information Acquisition and Security, Ministry of Industry and Information Technology, Harbin Engineering University, Harbin 150001, China; 4 College of Underwater Acoustic Engineering, Harbin Engineering University, Harbin 150001, China

**Keywords:** underwater acoustic channels, doubly spread, delay-Doppler-spread function, low-rank matrix recovery

## Abstract

In this paper, the estimation of overspread, i.e., doubly spread underwater acoustic (UWA) channels of strong dispersion is considered. We show that although the UWA channel dispersion causes the degeneration of channel sparsity, it leads to a low-rank structure especially when the channel delay-Doppler-spread function is separable in delay and Doppler domain. Therefore, we introduce the low-rank criterion to estimate the UWA channels, which can help to improve the estimation performance in the case of strong dispersion. The estimator is based on the discrete delay-Doppler-spread function representation of channel, and is formulated as a low-rank matrix recovery problem which can be solved by the singular value projection technique. Simulation examples are carried out to demonstrate the effectiveness of the proposed low-rank-based channel estimator.

## 1. Introduction

Due to multi-path propagation and time-varying nature, the underwater acoustic (UWA) channel is known to be doubly spread in delay and Doppler domain [1,2,3,4], which is also referred to as doubly selective in the literature [5,6,7]. To combat the effects of delay-Doppler spread in time-varying UWA channels, accurate estimation of the multi-path delay, Doppler frequency, and the channel gain is needed, which is a challenging task for high- speed UWA communications [8,9].

Channel estimation is usually performed with the aid of training signals. One well-known scheme is the least-squares (LS) estimator. However, due to lack of exploiting any prior information of the channel, LS estimators usually require many training signal measurements to achieve good estimation, especially when the number of unknown parameters is large [10]. Therefore, various channel estimation schemes have been proposed by considering the sparsity of channels, in which the technique of compressed sensing (CS) plays an important role [11]. For example, the orthogonal matching pursuit (OMP) and basis pursuit (BP) algorithms are adopted in [12] to estimate sparse doubly spread channel, which are shown to outperform the LS approach and subspace methods. It was further shown that BP has better performance than OMP in the UWA environment at the expense of much higher computational complexity [13]. In [2], a sparse channel estimation technique is developed based on the delay-Doppler-spread function representation of the channel. In [3], a computationally efficient two-stage sparse channel estimation technique is developed by parameterizing the amplitude variation and delay variation of each path with polynomial approximation. In a recent work [14], a sparse channel estimation technique has been proposed based on the Alamouti’s spacetime block coding with transmit diversity scheme in the form of two transmit antennas and one receiver.

The performance of the CS-based channel estimation relies highly on the sparsity of the channel. However, in practice, the sparsity assumption does not always hold for UWA channels due to the leakage effect and rich scattering environment [11,15]. In fact, different from the land-based radio channels which are known to be underspread, the UWA channels can tend to be overspread [16], which means that the UWA channels exhibit a strong dispersion/diffusion along delay and Doppler domain. The performance CS-based estimator can be significantly degraded due to the poor sparsity of overspread UWA channels [11,17,18]. Several techniques have been proposed to deal with the less sparse case. For example, the basis expansion [6,11,17] has been considered to enhance the sparsity of channels. In [7,17,18], some block/group compressed sensing techniques are introduced to fit the block sparsity structure of channels.

In this paper, the estimation of a dispersive overspread UWA channel is considered. By formulating the channel in a discrete matrix with respect to the delay-Doppler-spread function (called the delay-Doppler-spread matrix (DDSM) in this paper), we show that the channel dispersion gives rise to a useful low-rank structure of DDSM, especially when the channel delay-Doppler-spread function is separable in delay and Doppler domain. This low-rank structure reveals that although the overspread channel is not sparse in delay-Doppler domain, the DDSM can be determined by only a small number of singular values. Therefore, we introduce the low-rank criterion to estimate the overspread channels, which can help to improve the estimation performance in case of strong dispersive UWA channels. The estimator is formulated in a low-rank matrix recovery problem and is solved by the singular value projection (SVP) technique. Numerical experiments are carried out to demonstrate the effectiveness of the proposed low-rank-based channel estimator.

The rest of the paper is organized as follows. In Section 2, we give a discrete model of doubly spread channel-based on the delay-Doppler-spread function representation. In Section 3, we introduce the concept of underspread and overspread of wireless channels, and show the degeneration of sparsity in case of large dispersion spread. In Section 4, we show the low-rank structure of UWA channel and describe the proposed method. Finally, some computer simulation results are given to illustrate the behavior of the proposed method in Section 5, and the conclusions are drawn in Section 6.

*Notations:* Vectors are denoted by bold lower-case letters, while matrices use upper-case bold letters. C, R denote the set of complex and real numbers, respectively. $A^{T}$, $A^{H}$ and $A^{†}$ are the transpose, conjugate transpose and Pseudo inverse of A, respectively. vec(A) denotes the vectorization operator that stacks matrix A column by column. rank(•) are the rank operators, respectively. 0 denotes the zero matrix. ${∥A∥}_{2}$, ${∥A∥}_{F}$ and ${∥A∥}_{*}$ denote the $\ell_{2}$-norm, Frobenius norm and nuclear norm of A, respectively.

## 2. System Model

Consider signal x(t) propagating over a narrow-band time-varying UWA channel, the received signal y(t) can be written as

(1) y(t)=∫h(t,τ)x(t−τ)dτ+ω(t),

where h(t,τ) is the channel impulse response and ω(t) is additive noise. The delay-Doppler spreading function $S_{h}\left(\tau ,f\right)$, which represents the channel in terms of delay and Doppler frequency, is defined as the Fourier transform of h(t,τ)

(2) $$S_{h}\left(\tau ,v\right)=\int h\left(t,\tau \right)e^{-j2\pi vt}dt$$

where *v* is the variable in the Doppler domain. By combining (1) and (2), we have

(3) $$y\left(t\right)=\;\int \int \;S_{h}\left(\tau ,v\right)x\left(t-\tau \right)e^{j2\pi vt}d\tau dv+w\left(t\right)$$

We assume that the bandwidth and the time duration of the transmitted signal are *W* and *T*, respectively, which corresponds to a signal length of N=WT. Moreover, we assume that the channel has limited spreads in delay-Doppler domain, i.e., the delay spreads are within $\tau \in [0,\tau_{\max}]$, and Doppler spreads are within $v\in [-v_{\max}/2,v_{\max}/2]$. Then, by uniformly sampling the delay-Doppler space with Nyquist sampling rate (Δτ,Δv)=(1/W,1/T), we can obtain a discrete representation of (3) as [19]

(4) $$\begin{array}{c} y\left[n\right]=\sum_{l=0}^{L-1}\sum_{m=-M}^{M}S_{h}\left[l,m\right]e^{j2\pi mn/N_{0}}x\left[n-l\right]+\omega \left[n\right],\\ n=0,\ldots ,N_{0}-1. \end{array}$$

where $L=\lceil W\tau_{\max}\rceil +1$, $M=\lceil Tv_{\max}/2\rceil$ and $N_{0}=N+L-1$.

Collecting $N_{0}$ points of the received signal, we can rewrite (4) in a matrix form as

(5) $$y=A\mathrm{vec}[S_{h}]+\omega$$

where

$$y={\left[y\left[0\right],\ldots ,y\left[N_{0}-1\right]\right]}^{T}$$

$$\omega ={\left[\omega \left[0\right],\ldots ,\omega \left[N_{0}-1\right]\right]}^{T}$$

and $S_{h}\in C^{L\times 2M+1}$ is the DDSM with its (l,m)th element being $S_{h}\left[l,m\right]$.

$A\in C^{N_{0}\times L\left(2M+1\right)}$ in (5) is a block matrix of the form

$$A=[\Phi_{-M}X,\ldots ,\Phi_{M}X].$$

where $\Phi_{m}\in C^{N_{0}\times N_{0}}$ is a diagonal matrix given by $\Phi_{m}=\mathrm{diag}\left[e^{j2\pi m\cdot 0/N_{0}},\ldots ,e^{j2\pi m\left(N_{0}-1\right)/N_{0}}\right]$, and $X\in C^{N_{0}\times L}$ is a Toeplitz matrix whose first row and first column are given by $\left[x\left[0\right],0_{L-1}^{T}\right]$ and ${\left[x\left[0\right],\ldots ,x\left[N-1\right],0_{L-1}^{T}\right]}^{T}$, respectively.

Therefore, given measurement vector y and sensing matrix A, the doubly spread channel estimation problem has been transformed to the estimation of DDSM $S_{h}$ as shown in (5).

## 3. Overspread Channels

The doubly spread channel is commonly referred to be *underspread* if $\tau_{\max}v_{\max}\leq 1$, and *overspread* otherwise [16]. It is widely known that wireless radio channels are always underspread. This is because the channel delay and Doppler are both inversely proportional to the speed of light [16]. However, situation can be very different for UWA channels, since the speed of sound in water is much slower than the light speed. Specifically, we have $\tau_{\max}=d_{\max}/c$ and $v_{\max}=\nu_{\max}f_{c}/c$, where $d_{\max}$ is the maximum path length, $\nu_{\max}$ is the maximum relative velocity, $f_{c}$ is the carrier frequency and *c* is the speed of light or sound. If the channel is overspread, we need $d_{\max}\nu_{\max}f_{c}>c^{2}$. For radio communications, it is practically impossible since $c=3\times 10^{8}$ m/s and $f_{c}$ is on the order of $10^{6}∼10^{7}$ Hz. However, this can be true for UWA channel with $c=1.5\times 10^{3}$ m/s and $f_{c}=10^{2}∼10^{5}$ Hz.

Please note that under the overspread assumption, i.e., $\tau_{\max}v_{\max}>1$, we have that (2M+1)/T·L/W>1⇒L(2M+1)>WT=N. This means that the estimation problem of $S_{h}$ in (5) is under-determined. Thus, the classical LS estimator, i.e.,

(6) $$\begin{array}{c} {\hat{S}}_{h}=\arg\min_{S_{h}}{∥y-A\mathrm{vec}\left(S_{h}\right)∥}_{2}^{2}=A^{†}y \end{array}$$

may not give a satisfactory performance [20].

Numerous studies tackle the under-determined problem by considering the sparse property of the channel, where the technique of CS has been successfully applied. However, the CS-based estimator relies highly on the sparsity of the channel and the overspread channel may be of poor sparsity in delay-Doppler domain. Denoting *ℑ* as the support of $S_{h}\left(\tau ,v\right)$, i.e., $S_{h}\left(\tau ,v\right)=0$ for (τ,v)≠ℑ, then the area of its support |ℑ| can be used as a measurement of the dispersion spread of $S_{h}\left(\tau ,v\right)$. The overspread property of the underwater acoustic channel expresses the fact that the channel’s dispersion spread |ℑ| is relatively large. Hence the sparsity assumption may not directly hold for $S_{h}\left(\tau ,v\right)$ for overspread UWA channels, which motivates us to look for other criterion to estimate $S_{h}\left(\tau ,v\right)$ in case of large dispersion spread.

## 4. Proposed Method

### 4.1. Low-Rank-Based Estimator

Although the strong dispersive overspread UWA channels are not of good sparsity, they can enjoy a low-rank or approximately low-rank structure. Consider a doubly spread UWA channel under rich multi-path scattering environment, but tends to be dominated by a relatively small number of clusters of significant paths. Specifically, *P* dominant paths (clusters) are assumed with each dominant cluster formulated by many non-resolvable sub-paths which give rise to a relatively large dispersion spread. Please note that the well-known leakage effect [11] can also leads to a dispersion spread.

To show the low-rank structure of the UWA channel, we first consider the case that the delay and Doppler are separable in the spreading function, then $S_{h}\left(\tau ,v\right)$ can be expressed as

(7) $$S_{h}\left(\tau ,v\right)=\sum_{p=1}^{P}f_{p}\left(\tau \right)g_{p}\left(v\right)$$

where $f_{p}\left(\tau \right)$ and $g_{p}\left(v\right)$ describe the delay and Doppler profile of the *p*th dominant path, respectively. For sparse channel models considered in the literature, $f_{p}\left(\tau \right)$ and $g_{p}\left(v\right)$ are usually assumed to be of a ’sharp’ shape function with a small spread, such as the Dirac delta function or *sinc* function [10,11,16]. In this paper, we did not have such restriction, and the model in (9) generally takes the case of large dispersion spreads into account.

Following the discrete sampling model in (4), we can rewrite the DDSM as

(8) $$S_{h}=\sum_{p=1}^{P}f_{p}g_{p}^{T}$$

where vector $f_{p}={\left[f_{p}\left(0\right),\ldots ,f_{p}\left(\left(L-1\right)\Delta \tau \right)\right]}^{T}\in C^{L}$, and $g_{p}={\left[g_{p}\left(-M\Delta v\right),\ldots ,g_{p}\left(0\right),\ldots ,g_{p}\left(M\Delta v\right)\right]}^{T}\in C^{2M+1}$. Then it is obvious that $f_{p}g_{p}^{T}$ is a rank-one matrix, and thus the spreading function matrix $S_{h}$ is a low-rank matrix with its rank no more than *P*. i.e.,

$$\mathrm{rank}(S_{h})\leq P$$

Please note that the low-rank property of $S_{h}$ is independent of the shape and dispersion spread of the function $f_{p}\left(\tau \right)$ and $g_{p}\left(v\right)$ in this case.

In practice, when the spreading function may not perfectly separable in delay and Doppler domain, then the matrix $f_{p}g_{p}^{T}$ is generally not a strictly rank-one matrix. However, since the dispersion due to scattering or leakage effect tends to independent in delay and Doppler domain, the DDSM can still be well approximated by a low-rank matrix. We will further show the approximate low-rank property through numerical examples in the simulation section. Moreover, since

(9) $$S_{h}\left(\tau ,v\right)=\sum_{(\tau_{i},v_{j})\in ℑ}S_{h}\left(\tau ,v\right)\delta \left(\tau -\tau_{i}\right)\delta \left(v-v_{j}\right)$$

then it is easy to verify

(10) $$\mathrm{rank}(S_{h})\leq |ℑ|$$

which means that the rank of $S_{h}$ is no more than the sparsity of $S_{h}$. In fact, *rank* describes the 2-D structured sparsity of a matrix, which exploits the correlation between columns and rows within a matrix. While for the classic sparsity measurement, aka, $l_{0}$ or $l_{1}$ norm, is essentially a 1-D sparsity measurement which ignores the 2-D structure in the original matrix. Since the UWA channel is known to have a clustering structure and the DDSM is 2-D in nature, we propose to use low-rank criterion to estimate the DDSM. Please note that for certain extreme cases where the UWA is not low-rank, however, is not the scope of this paper.

To exploit the low-rank property, one can obtain an estimate of $S_{h}$, denoted as ${\hat{S}}_{h}$. Mathematically, it can be expressed as

(11) $$\begin{array}{cc} {\hat{S}}_{h} & =\arg\;\min_{S_{h}}\;\mathrm{rank}(S_{h})\\ s.t. & \;∥y-A\mathrm{vec}\left(S_{h}\right){∥}_{2}^{2}\leq \varepsilon^{2} \end{array}$$

where ε measures signal reconstruction errors.

Although the optimization problem (11) is simple in form, it is difficult to be solved due to the non-convexity and discrete nature of the rank function. Fortunately, many techniques have been developed for the low-rank matrix recovery framework.Following the idea of SVP framework [21,22], we reformulate the low-rank overspread channel estimation problem as

(12) $$\begin{array}{cc} {\hat{S}}_{h} & =\arg\;\min_{S_{h}}\{J\left(\mathrm{vec}\left(S_{h}\right)\right)=∥y-A\mathrm{vec}\left(S_{h}\right){∥}_{2}^{2}\},\;s.t.\;\mathrm{rank}\left(S_{h}\right)\leq P \end{array}$$

The solution of (12) can be achieved by SVP at each iteration. In particular, in the *i*th iteration, the current result ${S_{h}}^{i}$ is projected onto a low-rank matrix $S_{h}^{i*}$, which is defined as $S_{h}^{i*}=\mathrm{SVP}\left(S_{h}^{i}\right)=\sum_{p=1}^{P}u_{p}s_{p}v_{p}$, where ${\left\{s_{p}\right\}}_{p=1}^{P}$ is the *P* most significant singular values of $S_{h}^{i}$. The SVP algorithm is shown in Algorithm 1 [21,22].

**Algorithm 1:** The SVP algorithm.**Input: y, A** **Initialization: P, $S_{h}^{0}=0$, i = 1** **Repeat:** **1.**$\mu \leftarrow -\frac{d_{i-1}^{H}d_{i}}{d_{i}^{H}\left(2A^{H}A\right)d_{i}},\mathrm{where}\;d_{i}\mathrm{is}\;\mathrm{the}\;\mathrm{gradient}\;\mathrm{of}\;J\left(\mathrm{vec}\left(S_{h}^{i}\right)\right)$ **2.**$\mathrm{vec}\left({S_{h}}^{i}\right)\leftarrow \mathrm{vec}\left({S_{h}}^{i}\right)+\mu d_{i}$; **3.**${S_{h}}^{i}\leftarrow \mathrm{SVP}\left({S_{h}}^{i}\right)$; **4.**i←i+1; **Until   convergence** **Output:**$S_{h}$

It should be noted that we only use the SVP method to demonstrate the validity of the proposed low-rank estimator, and it could be replaced by other suitable low-rank matrix recovery techniques.

### 4.2. Complexity

The computational complexity of the proposed estimator depends on the techniques used for solving the low-rank matrix recovery problem. For example, the SVP solver approximately has a complexity order of $O(\left(\max\left(L,2M+1\right)P^{2}+N_{0}^{2}L\left(2M+1\right)\right)N_{iter})$, where $N_{iter}$ is the number of iterations [22]. For sparsity-based methods, for instance the OMP method, the complexity is $O(\left(N_{0}L\left(2M+1\right)+N_{0}\right)N_{iter})$ [15]. We can see that the low-rank-based method is generally has more computational burden than the sparsity-based methods. However, as we will see in the simulation section, the low-rank-based method may outperform the sparsity-based method in case of overspread UWA channels.

## 5. Simulation Results and Performance Comparison

In this section, we illustrate the performance of the proposed method through numerical examples. The time-varying UWA channel is generated with statistical underwater acoustic channel model proposed in [1]. Specifically, the time-domain channel impulse response h(t,τ) is generated with an observation period of T=2 s and sampling rate of W=1.6 KHz. Then we can obtain the spreading function $S_{h}\left(v,\tau \right)$ by applying the Fourier transform of h(t,τ) with respect to *t*. We consider $S_{h}\left(v,\tau \right)$ is limited in delay domain as τ∈[0,50] ms and Doppler domain as v∈[−20,21] Hz, which corresponds to *L* = 50 ms × 1.6 KHz = 80 and 2M + 1 = 2 s × 41 Hz = 81.

**Figure 1:**
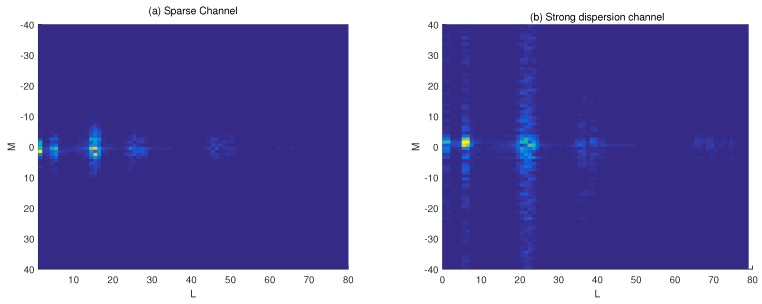
Channel DDSM generated by the model in [1]. (**a**) Sparse Case; (**b**) Dispersion Case.

**Figure 2:**
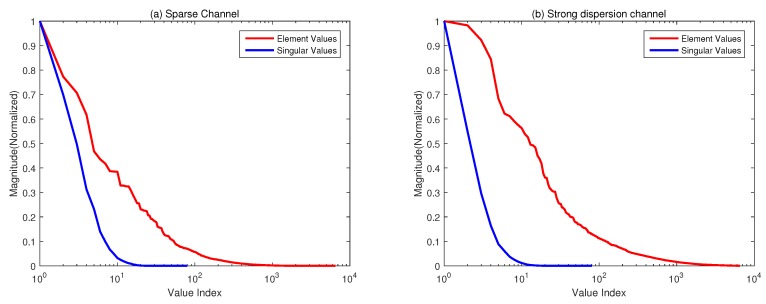
Comparison between the matrix values (magnitude) and singular values of DDSM. (**a**) Sparse Case; (**b**) Dispersion Case.

Both the underspread (sparse) and overspread (strong dispersion) channel models are considered in the first example. The DDSMs of the UWA channel are shown in Figure 1a,b (with Doppler and time delay normalized with respect to the frequency sampling rate Δv=1/T and Δτ=1/W, respectively). To show the low-rank property of the channel, we plot the singular values of DDSM (normalized with the maximum value) as shown in Figure 2. For comparison, we also show the sparsity of DDSM by plotting the absolute values of each element in DDSM in descending order (normalized with the maximum value). We can see that the number of dominant singular values of $S_{h}$ is smaller than that of its elements, especially in case of the strong disperse channel.

**Figure 3:**
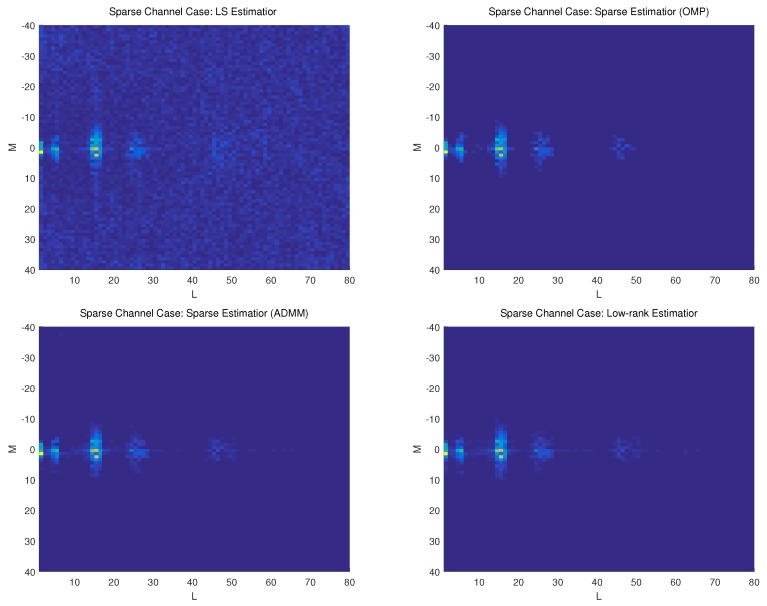
DDSM estimated by different estimators under sparse channel case. (**a**) LS method; (**b**) Sparsity-based Method; (**c**) Low-rank-based method.

**Figure 4:**
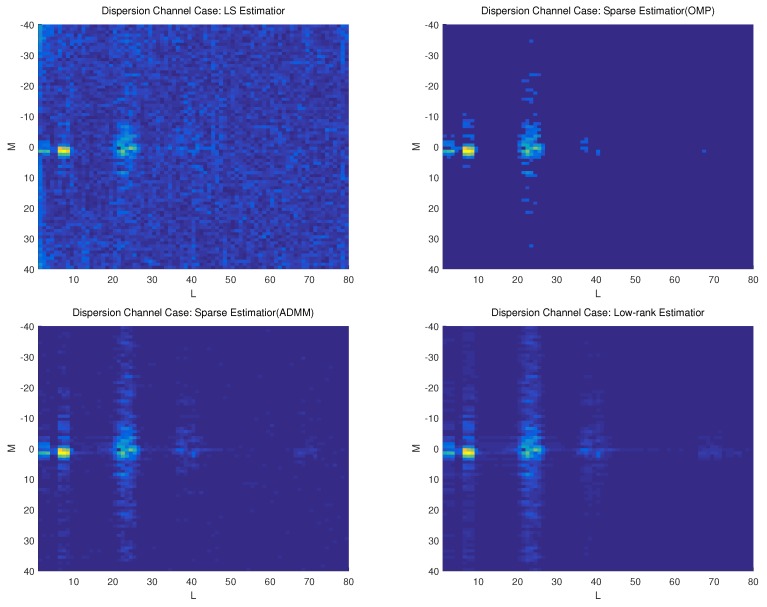
DDSM estimated by different estimators under dispersion channel case. (**a**) LS method; (**b**) Sparsity-based Method; (**c**) Low-rank-based method.

In the second example, we estimate both the sparse and dispersion UWA channel through a training signal length of N=WT=3200. The signal-to-noise ratio (SNR) is 20 dB. Besides the proposed low-rank estimator, we also consider the classic LS estimator and the sparsity-based methods for performance comparison. The orthogonal matching pursuit (OMP) algorithm [12,23,24] and the alternating direction method of multipliers (ADMM) [25] is adopted to solve the sparsity-based estimator. Figure 3 and Figure 4 show the 2-D plots of the estimated DDSM of by different methods under sparse and dispersion channel cases, respectively.

We can see from Figure 3 and Figure 4 that the LS-based method leads to many pseudo paths as well as limited accuracy under both sparse and dispersion channel cases. That is because LS method does not exploit either sparsity or low-rank property of the DDSM. The OMP and ADMM methods work well for the sparse channel case due to its sparsity exploitation. For the dispersion case, however, they suffer from performance degradation due to the poor sparsity of the overspread channel. The proposed low-rank estimator can achieve a comparable performance for the sparse channel case, and better performance than the compared methods for the dispersion channel case.

In the third example, we compare the normalized mean squared error (NMSE) performance of the considered estimators. The NMSE is defined as

$$\mathrm{NMSE}=E\{\frac{∥S_{h}-{\hat{S}}_{h}{∥}_{F}^{2}}{∥S_{h}{∥}_{F}^{2}}\}.$$

**Figure 5:**
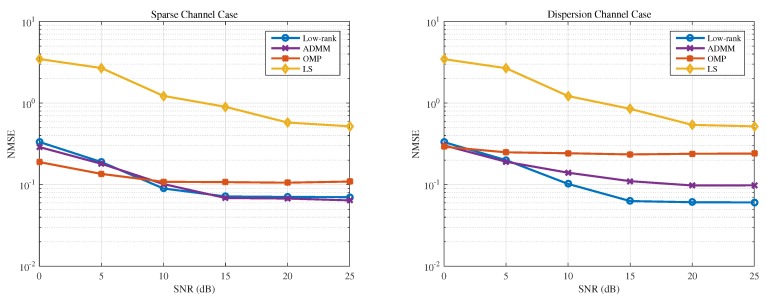
NMSE comparison versus SNR.

The NMSE performance versus different SNR is shown in Figure 5. We can see that the proposed low-rank-based estimator has similar performance to the ADMM method in case of sparse channel, and outperforms the two sparsity-based estimators and the LS estimator in case of dispersive channel under relatively higher SNRs.

**Figure 6:**
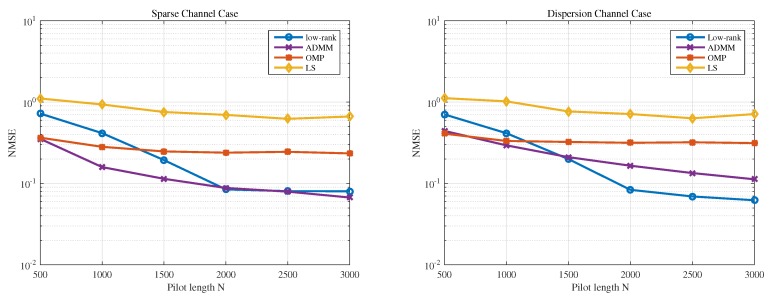
NMSE comparison versus Signal length *N*.

In the fourth example, we investigate the estimation performance versus signal length *N*. The SNR is fixed to 20 dB, and *N* varies from 500 to 3000. As shown in Figure 6, the proposed Low-rank-based method again outperforms the other compared methods in case of dispersive channel under moderate or large number of training signal.

**Figure 7:**
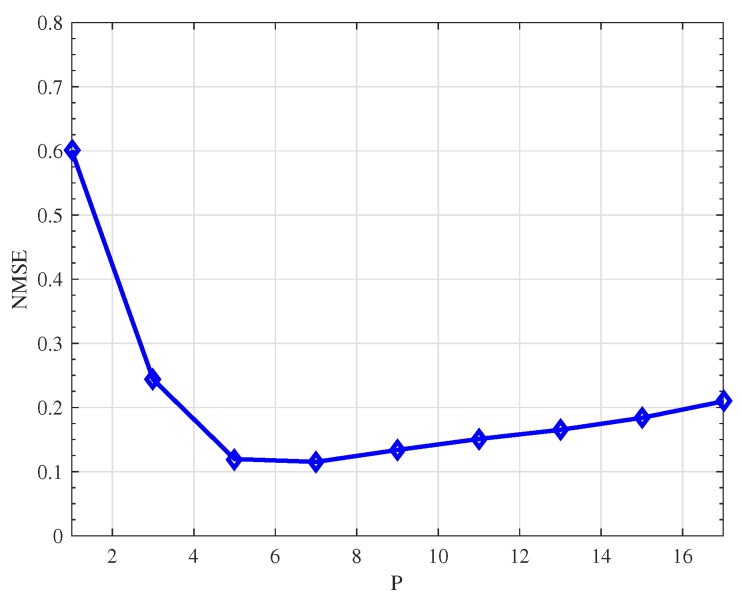
NMSE comparison versus parameter *P*.

Finally, we investigate the impact of user parameters in the proposed method under. The key parameter in SVP algorithm is the rank projection parameter *P*. The NMSE performance of the proposed method versus parameter *P* is shown in Figure 7. We can see that the proposed method achieves the best performance when *P* is near 5 to 9, which is approximately the number of the significant singular values of the DDSM. Moreover, we can also indicate from Figure 7 that it is better to overestimate rather than underestimate *P* when the exact rank of the DDSM is not known in practice.

## 6. Conclusions

In this paper, the estimation of a dispersive overspread UWA channel is considered. We formulate the channel input–output relationship in a discrete delay-Doppler-spread function representation. Then we show that although the channel dispersion causes the degeneration of channel sparsity, it gives rise to a useful low-rank structure, when the channel delay-Doppler-spread function is separable in delay and Doppler domain. This low-rank structure reveals that the matrix representation of delay-Doppler-spread function can be determined by only a small number of singular values. Therefore, we introduce the low-rank criterion to estimate the overspread channels, which can help to improve the estimation performance in case of strong dispersive UWA channels. The estimator is formulated in low-rank matrix recovery problem and is solved by the SVP technique. Simulation examples are carried out to demonstrate the effectiveness of the proposed low-rank-based channel estimator. In future work, it is interesting to further consider a joint sparse and low-rank criterion for UWA channel estimation.
